# A Large Retroperitoneal Hydatid Cyst: A Cause of Recurrence and What to Consider in Their Management

**DOI:** 10.1155/crdi/4699254

**Published:** 2025-11-03

**Authors:** Svetlana Shumarova, Manol Sokolov

**Affiliations:** ^1^Department of Surgery, Medical University, 1 Georgi Sofijski Blvd, Sofia 1431, Bulgaria; ^2^University Hospital “Aleksandrovska”, 1 Georgi Sofijski Blvd, Sofia 1431, Bulgaria

**Keywords:** echinococcus, hydatid cyst, retroperitoneal

## Abstract

Retroperitoneal hydatid cyst caused by *Echinococcus granulosus* is a rare clinical condition that can occur primarily or synchronously with other location. We present a 47-year-old man with a recurrent retroperitoneal echinococcal cyst, complaining of pain in the right lumbar region. The diagnosis was established by ultrasound and computed tomography; additionally a partial cystectomy was performed. Retroperitoneal echinococcal cysts are often initially interpreted as retroperitoneal tumors, except in cases with a history of echinococcosis, which always leaves a degree of doubt regarding the presence of a recurrence or the development of a new cyst. In the present case, the surgical approach was the appropriate choice, followed by antihelminthic therapy. Only the combination of these two approaches can prevent the troublesome complication of echinococcosis recurrence.

## 1. Introduction

Echinococcosis is a zoonosis caused by a tapeworm infection from the genus Echinococcus, with *Echinococcus granulosus* causing the cystic form and Echinococcus multilocularis causing the alveolar form. The main annual incidence from 1997 to 2020 throughout Europe was 0·64 cases per 100,000 people [1]. The liver is the most common site of involvement (65%), followed by the lungs (25%), but other locations can also be affected, including the bones, spleen, central nervous system, and heart [2], and more rarely the retroperitoneal space [3, 4]. Retroperitoneal echinococcosis can sometimes be misinterpreted as a tumor mass, which makes accurate preoperative diagnosis essential to determining the appropriate individualized therapeutic approach. This depends on factors such as the size of the cyst, its proximity to adjacent structures, and the patient's overall condition. In this article, we report a case of recurrence of a large retroperitoneal echinococcal cyst.

## 2. Case Presentation

**Figure 1:**
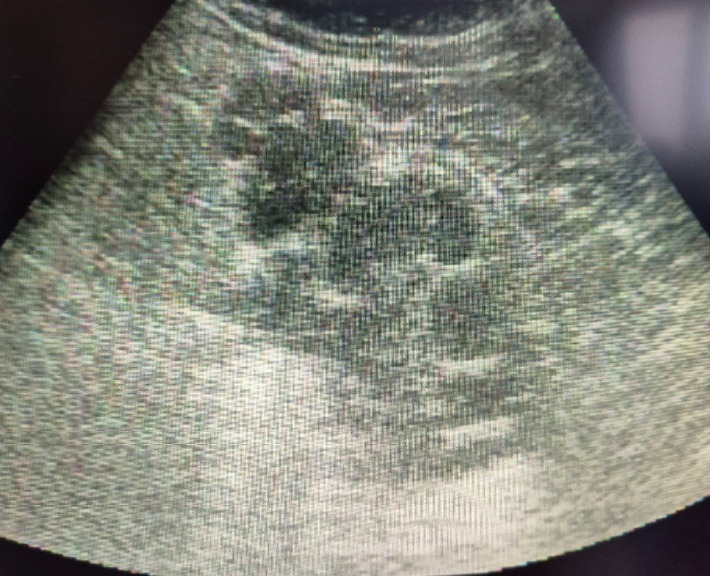
Ultrasound image of a hydatid retroperitoneal cyst with multiple scolexis.

**Figure 2:**
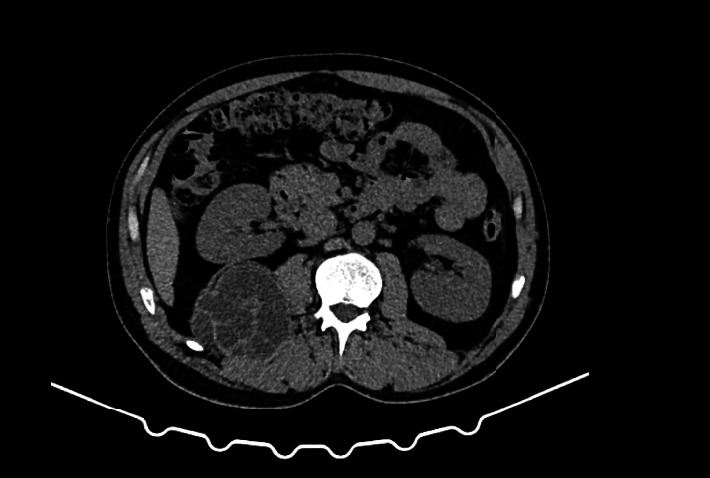
CT image of a retroperitoneal hydatid cyst on the right site.

**Figure 3:**
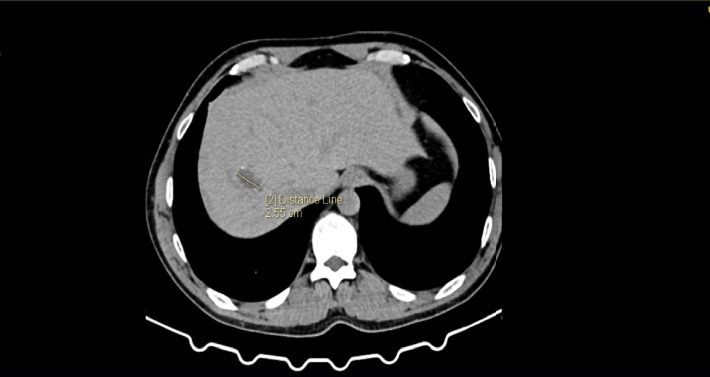
CT scan shows the site of the hepatic echinococcectomy 3 years after the first surgery.

**Figure 4:**
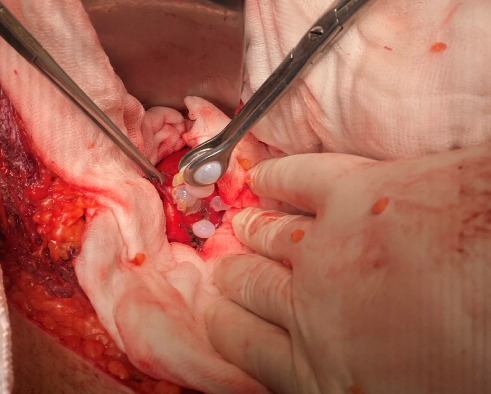
Retroperitoneal hydatid cyst with an open capsule filled with scolexis.

**Figure 5:**
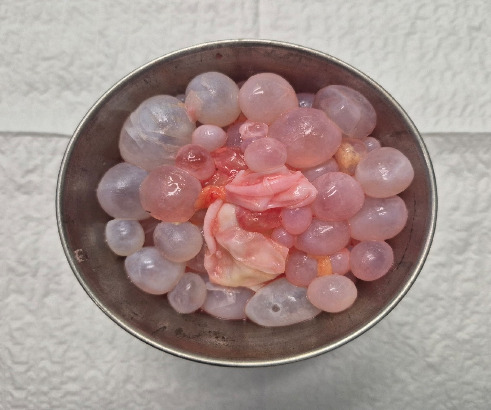
All scolexis removed from cyst cavity.

A 47-year-old man was admitted for evaluation with complaints of pain and a sensation of heaviness in the right lumbar region for several months. The patient had a history of surgery 3 years earlier for a hepatic cyst in Segment VIII and a retroperitoneal echinococcal cyst. Both cysts were treated with a scolicidal solution. Capitonnage of the hepatic cyst was performed, while the capsule of the retroperitoneal cyst was not completely removed. The patient did not follow the physician's recommendation to take albendazole after the first surgery. Three years later, with the onset of symptoms, an ELISA test for hydatid disease was performed, which turned out to be positive. Laboratory tests showed no abnormalities. Abdominal ultrasonography (US) revealed a cystic formation in the right retroperitoneal space, containing multiple oval hypoechoic structures, suspected to be a hydatid cyst (Figure 1). Computed tomography (CT) demonstrated a multicystic oval lesion in the right retroperitoneal space measuring approximately 15 cm in diameter, adjacent to the quadratus lumborum and psoas major muscles as well as the thoracolumbar fascia. Ventrally, it lay against the posterior surface of the right kidney, compressing it anteriorly (Figure 2). In Segment VIII of the liver, at the site of the previous cyst, a hypodense lesion was observed with peripheral calcifications measuring approximately 29 × 14 mm in the axial plane, without septations or daughter cysts (Figure 3). The patient underwent surgery via a laparotomy approach. The ascending colon and right kidney were subsequently mobilized. Posterior to the kidney, a cystic formation was visualized measuring approximately 15 cm in diameter with a dense fibrous capsule (Figure 4), containing multiple chambers and scolices within the lumen (Figure 5). The surgical field was isolated with gauze pads soaked in 25% hypertonic NaCl solution, after which the cavity was repeatedly irrigated with the same solution and its contents evacuated. All interchamber spaces were bluntly opened, and the capsule was almost entirely removed, and a small portion of the posterior wall was left, due to the risk of injury to the renal vessels. Drains were placed in the cyst bed, which did not produce any output in the postoperative period. The patient was discharged on the fifth postoperative day. Histological analysis of the capsule confirmed a hydatid cyst, and an antiparasitic therapy was recommended.

**Figure 6:**
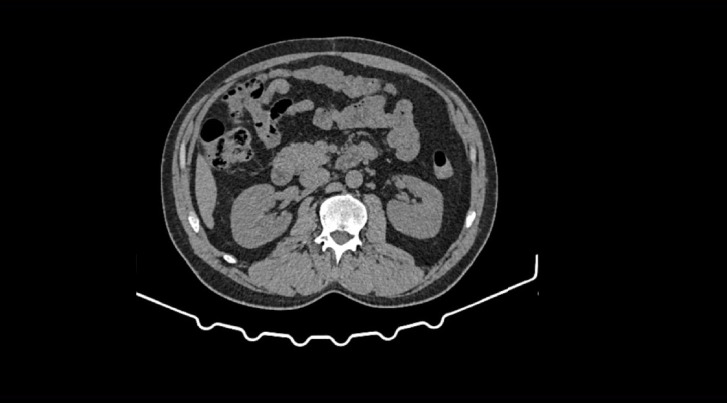
CT scan 8 months after surgery for the retroperitoneal cyst shows normal positioning of the right kidney and no evidence of recurrence.

Three cycles of treatment with albendazole 400 mg twice daily for 28 days, with a 14-day break between cycles, were administered. Eight months after surgery, follow-up abdominal CT showed the persistent hepatic area in Segment VIII without changes. No cystic lesions were detected in the right retroperitoneal region at the site of the previous surgical intervention (Figure 6).

## 3. Discussion

There is a growing number of case reports describing primary retroperitoneal cysts, which aim to enhance the available information and provide greater utility for appropriate therapeutic management. The localization and nonspecific symptoms of this type of cyst often lead to delayed or incorrect diagnosis. They are associated with a high risk during surgical treatment due to their proximity to vital anatomical structures. Secondary echinococcal cysts most commonly arise as a result of a rupture or leakage, dissemination of parasitic material, or formation of new cysts. Proper management includes adjunctive pre- or postoperative antiparasitic therapy with albendazole. We present this case to emphasize the importance of this combined approach to prevent one of the most common complications—recurrence—as illustrated in our case.

A recent large retrospective analysis was conducted by He J et al. [5], including 1257 cases at a single hospital between 2012 and 2019. Of these, 121 cases (9.63%) had confirmed retroperitoneal echinococcosis. Primary retroperitoneal echinococcosis is often misinterpreted as a malignant tumor [6], making it important for surgeons to be aware of in differential diagnosis. In our case, the initial interpretation was of a hydatid cyst, although the patient had a history of prior echinococcal surgery.

Hydatid cysts can occur at any age, with the most common complaints being a sensation of heaviness and pain at the site of the cyst, which are related to its size and compression of adjacent structures. Often, the first diagnostic tool available is US, which reveals a cystic formation with multiple septa. According to the World Health Organization Informal Working Group on Echinococcosis (WHO-IWGE), ultrasound classification includes five stages that help guide the operator regarding the activity of the echinococcus [7], which play a crucial role in determining the subsequent therapeutic approach. It would be beneficial for patients with ultrasound signs of active disease (Stages CE1, CE2, and partly CE3) to be considered for preoperative antihelminthic therapy. This approach can reduce the risk of intraperitoneal dissemination during surgery and postoperative recurrence. Many authors have recommended preoperative treatment with albendazole [8–12], while others have used postoperative therapy alone [13, 14], both aiming to reduce recurrence to zero [8–12, 14] (Table 1). This case highlights the critical importance of patient compliance with postoperative anthelmintic therapy, as the patient's nonadherence likely contributed to the disease recurrence. Ultrasound cannot provide sufficient information regarding the involvement of adjacent structure near a retroperitoneal cyst. In such cases, CT and MRI are helpful, with MRI considered more effective for interpreting the structural characteristics of the cysts. In addition, tests for specific IgG and IgG4 against recombinant *E. granulosus* antigen P29 (rEg.P29) can serve as an auxiliary method to identify cyst activity and determine the optimal therapeutic plan for cystic echinococcosis [15].

The occurrence of cysts in unusual locations may be secondary, resulting from cyst rupture, according to some authors, or intraoperatively due to improper handling and treatment of the cavity. Analyzing the clinical case data presented in Table 1 for primary retroperitoneal cysts, makes it evident that a large proportion is located on the left side [3, 8, 9, 11–14], suggesting a likely lymphogenous implantation via the vessels of the gastrointestinal tract. Our case provides further evidence for the possibility of simultaneous implantation through both the portal system and lymphatic routes, as demonstrated by the synchronous localisation of two cysts—in the liver and the right retroperitoneal space.

The gold standard for the treatment of echinococcal cysts is a combination of albendazole therapy and surgical intervention—preferably total cystectomy [4, 10, 11, 13]. However, when there is a risk of injury to vital structures, partial cystectomy is a viable option, yielding good outcomes without recurrence [8, 9, 12, 14]. Surgical treatment without albendazole intake increases the risk of recurrence, as demonstrated in our case. The preferred approach to the abdominal cavity is laparotomy, due to the large size of the cysts and the retroperitoneal location, which requires thorough exploration of the abdominal organs, and adequate visualization. Nevertheless, with the advancement of minimally invasive surgery, successful laparoscopic partial cystectomy without recurrence has also been reported by Slavu et al. [12].

## 4. Conclusion

Adequate treatment of large, solitary echinococcal cysts, regardless of their location, involves a a combination of antihelminthic therapy and surgical intervention, with the goal of reducing the recurrence rate to zero. The absence of antihelminthic therapy carries a risk of recurrence, as demonstrated in the reported case.

## Supporting Information

Table 1: Analysis of clinical cases and review of the literature.
